# Measurement of the Adipose Stem Cells Cell Sheets Transmittance

**DOI:** 10.3390/bioengineering8070093

**Published:** 2021-07-02

**Authors:** Jun Ochiai, Yutaka Niihara, Joan Oliva

**Affiliations:** Emmaus Medical, Inc., 21250 Hawthorne Blvd., Suite 800, Torrance, CA 90503, USA; jochiai@emmauslifesciences.com (J.O.); yniihara@emmauslifesciences.com (Y.N.)

**Keywords:** adipose stromal cells, stratified cell sheet, transmittance, harvesting

## Abstract

In the field of cell therapy, the interest in cell sheet technology is increasing. To determine the cell sheet harvesting time requires experience and practice, and different factors could change the harvesting time (variability among donors and culture media, between cell culture dishes, initial cell seeding density). We have developed a device that can measure the transmittance of the multilayer cell sheets, using a light emitting diode and a light detector, to estimate the harvesting time. The transmittance of the adipose stromal cells cell sheets (ASCCS) was measured every other day as soon as the cells were confluent, up to 12 days. The ASCCS, from three different initial seeding densities, were harvested at 8, 10, and 12 days after seeding. Real-time PCR and immunostaining confirmed the expression of specific cell markers (CD29, CD73, CD90, CD105, HLA-A, HLA-DR), but less than the isolated adipose stromal cells. The number of cells per cell sheets, the average thickness per cell sheet, and the corresponding transmittance showed no correlation. Decrease of the transmittance seems to be correlated with the cell sheet maturation. For the first time, we are reporting the success development of a device to estimate ASCCS harvesting time based on their transmittance.

## 1. Introduction

In the field of regenerative medicine, different approaches are used to treat patients with stem cells, such as 3D printing of “organs”, injection of the cells in the damaged organ, or the formation of a multilayer cell sheet that could be transplanted on organs. To increase the curative properties and the survival of the stem cells, it was shown in few publications that the grafting of the stem cells on the organs had a higher viability and a higher efficiency to improve the recovery of the damaged organs [1,2]. Regarding the cell sheet technology for grafting, different types of scaffolds were used for its development such as amniotic membrane [3], fibrin gel [4], hyaluronan hydrogel [5], collagen [6], thermo-responsive polymers [7], or no scaffold [8]. Formed cell sheets allow the harvesting of a complex structure to damaged organs areas. It was used with success on different animal models, organs, and patients [9,10,11]. Particularly, scaffold-free system for fabrication of three-dimensional (3D), multilayered cell sheet has attracted a lot of attention due to its curative effectiveness upon transplantation [12,13]. Traditional methodologies to form multilayered cell sheets are either to stack multiple monolayer cell sheets, each of which is cultured upon thermo-responsive polymer [14,15], or to apply magnetic force to accumulate magnetite-incorporated cells [16,17]. Although these methods have successfully formed multilayered cell sheets, there are potential limitations including specialized pre-treatment of culture dishes and cells, which could make the manufacturing process complex and costly. To be mechanically harvested, a cell sheet must possess physical integrity that is strong enough to retain the sheet structure when lifted from the cell culture dish. Maturity of cell sheets’ cell-to-cell connection, which is the origin of strong integrity and an essential indicator for the appropriate timing of harvesting, is dependent on multiple factors including types of cells, culture dish, initial cell density, composition of culture media, and the capability of cell proliferation [18]. In a conventional monolayer cell sheet engineering method, the determination of the harvest point is relatively simple since the cell sheets are harvested as soon as the cells reach confluence to form a monolayer [19]. In contrast, in a multilayer cell culturing system, cells develop further into hyperconfluent state as they grow three dimensionally, thus making it hard to predict whether a cell sheet has reached full maturation where mechanical harvesting is achievable. The manipulator needs to have some experience through careful observations of cell sheets’ appearance under microscope, which could be associated with, sometimes involving trial-and-error process, the right time for harvesting. Cell sheets fabricated from adipose stromal cells are reported to remain healthy as long as three weeks [18], or 32 days in our laboratory (data not shown), which shows the necessity of a means to evaluate the maturity of the cell sheet noninvasively and provide an objective measure of harvest time in a prolonged culturing period. It is known that depending on the ASC’s degree of development into cell sheets, the transparency of the cell sheets will vary [8]. As a cell sheet expresses interconnecting proteins and develops strong cell-to-cell connection, it will reduce the transmittance of the light and this decrease of the transmittance could be used, as a non-invasive methodology, to assess the maturity and thus estimate the harvesting time. Transplantable human cornea is one example where transparency becomes a crucial criterion for quality evaluation of sheet-like structured tissue. Before transplantation, the transparency of the cornea is assessed with a gross observation of the cornea [20,21]. Transparency estimation is based on the observer’s experience, and not from specific physical or optical criteria, that could be used as a standard. As demands for objective quality control standards increases, some publications reported the development of portable devices to measure the transparency of corneas [22,23,24,25] and some companies have developed instruments to study surface roughness, thickness of the objects/tissues. The same principle could be applied to cell sheet engineering.

Previously, our lab reported a successful fabrication of multilayered cell sheets using adipose stromal cells (ASC), free of scaffold, polymers, or external devices that aid formation and harvest of cell sheets [8,15,26,27]. ASC was chosen for its abundant availability with high accessibility compared to other types of mesenchymal stem cells, high proliferation nature, and multi-lineage differentiation potential [15,26,27]. It is essential for the cell sheet fabrication process to assess the maturity of samples in an objective and noninvasive approach. Although the application of optical coherence tomography (OCT) shows one possible way to achieve a noninvasive methodology to study the maturity of a cell sheet [28], the cost and size of the instrument, as well as the complexity of the technology have been a barrier for wide accessibility [29]. In this study, the laboratory decided to develop a simple, low-cost, and noninvasive device to measure the transmittance of undifferentiated multilayer ASC cell sheets as an indicator of its maturity and an estimation of the harvesting time that could be used in quality control.

## 2. Materials and Methods

### 2.1. Cell Culture

Human adipose stromal cells (hASC) were purchased from RoosterBio, Inc. (RoosterBio, Inc., Frederick, MD, USA). Human ASC was used for the following experiments. hASC were expanded up to passage 4, in T75 flask (USAScientific, Ocala, FL, USA), using RoosterNourish-MSC-CC culture media (RoosterBio, Inc., Frederick, MD, USA).

### 2.2. Engineering of the ASC Cell Sheets (ASCCS)

Different initial cell seeding density was used to engineer cell sheets: 2.24 × 10^4^, 5.6 × 10^4^, 10.2 × 10^4^ ASC per cm^2^ were seeded per 35 mm culture dish (Corning, Corning, NY, USA). The ASC were cultured with RoosterNourish-MSC-CC culture media (RoosterBio, Inc., Ballenger Creek, MD, USA). The culture media was replaced every 2 days, up to 12 days from the initial seeding day.

### 2.3. Differentiation of the ASC

Adipose stromal cells were seeded at 7 × 10^4^ cells/cm^2^ in a 12 well plate (Corning, Corning, NY, USA). The day after seeding, ASC were treated with the MesenCult™-ACF Chondrogenic Differentiation Kit (Stem Cell, Vancouver, Canada) and Osteomax-XF Differentiation Medium (Millipore-Sigma, Burlington, MA, USA). Chondrocyte cells were stained with the Alcian Blue Stain kit (Bioquochem, Llanera, Spain) and the osteoblast cells were stained with the Alizarin Red Stain kit (Millipore-Sigma, Burlington, MA, USA).

### 2.4. RNA Extraction and cDNA Synthesis

RNA from the ASCCS at 12 days and isolated ASC were extracted using the RNeasy Plus Mini Kit (Qiagen, LLC, Hilden, Germany). Synthesis of cDNAs was performed with 1 μg total RNA, and random hexamer primers using the High-Capacity cDNA Reverse Transcription Kit (Life Technologies, Carlsbad, CA, USA).

### 2.5. Real-Time PCR

Quantitative Real-time PCR were performed on the QuantStudio™ 3 System (ThermoFisher, Waltham, MA, USA). Real-time PCR experimental condition was as follows: an initial activation step at 95 °C for 10 min followed by 40 cycles of amplification that consisted of a denaturation step at 95 °C for 10 s, an annealing step (35 s at 60 °C), and finally, an extension step (15 s at 72 °C). Amplification efficiency was estimated by plotting a standard curve, using appropriate serial dilutions of cDNA samples for the genes of interest and housekeeping gene. The 2−ΔΔCT method was used to compare the relative level of expression of mRNA. The primers used for the real-time PCR are listed in the Table 1.

### 2.6. Genomic Extraction and Estimation of Cell’s Number per ASCCS

Because of the difficulty to dissociate the cells from the multilayer cell sheet (data not published and [30]) to obtain single cells, the number of cells was estimated by isolating total genomic DNA from entire cell sheets and compared to the quantity of genomic DNA from a determined number of isolated ASC. Genomic DNA, from isolated ASC and multilayer cell sheets, was isolated with the Wizard Genomic DNA purification Kit (Promega, Madison, WI, USA), following the manufacturer protocol. After harvesting the cell sheets, the ASCCS were “dissociated” with silica beads (Benchmark Scientific, Sayreville, NJ, USA), using Bead Mill 24 Homogenizer (Fisher Scientific, Hampton, NH, USA). Isolated single ASC were counted with trypan blue to estimate the quantity of double strand DNA per cell, using QuantiFluor dsDNA System (Promega, Madison, WI, USA).

### 2.7. Hematoxylin and Eosin Staining

Engineered cell sheets were fixed in 10% neutral buffered formalin and embedded in paraffin. Tissue sections were then stained with Hematoxylin and Eosin (H&E) (Bioquochem, Llanera, Spain). A Leica DMi1 was used to record the pictures (Leica, Wetzlar, Germany).

### 2.8. Immunocytochemistry Staining

Engineered cell sheets were fixed in 10% neutral buffered formalin and embedded in paraffin. Tissue sections were used for immunofluorescent staining with CD19, CD73 (NovusBIo, CO, USA), CD29, HLA-A, HLA-DR (Abcam, MA, USA), Oct3/4 (Novus Biologicals LLC., Littleton, CO, USA). Alexa Fluor 488 donkey anti-rabbit conjugated secondary antibodies and Alexa Fluor 488 donkey anti-mouse conjugated secondary antibodies (Invitrogen, Carlsbad, CA, USA) were used. Propidium iodide (Invitrogen, Carlsbad, CA, USA) was used to stain nuclear DNA. A EVOS M5000 microscope was used to analyze the slides (Invitrogen, Carlsbad, CA, USA).

### 2.9. Device to Measure the Transmittance of the ASCCS

The device is composed of a flat sample stage where the cell culture dish is placed, and light source (LED) placed above the sample stage. A light collimation unit consisting of two plano-convex lenses (Newport Corporation, Irvine, CA, USA) (KPX576, diameter = 25.4 mm, focal length = 25.4 mm) was also incorporated to reduce the effect of stray light. The LED used is a general-purpose white LED that emits phosphor-converted white light, which typically has broad spectral power distribution with a sharp peak at 450–475 nm for blue color and a flatten peak at 560–600 nm for yellow light. The light from LED used is not polarized. The light emitted from the LED first goes through an aperture that is located 5 mm away from the point of emission. The light subsequently travels through the collimation system by which stray light is reduced as well as the light is condensed on the sample surface. The detector used was OPT101 monolithic photodiode (Texas Instruments, Dallas, Texas). The sample stage is a motorized, belt-driven x-y linear stage which can be controlled electronically for the consistency in sample positioning. The components of the stage were 3D-printed and the driving unit was composed of a GT2 timing belt (FYSETC, pitch = 2 mm, width = 6 mm) and 28BYJ-48 stepper motor (ELEGOO). The stage was controlled by Arduino Uno R3 (Arduino, https://store.arduino.cc/usa/, accessed on 5 June 2021) microcontroller through the motor driver modules so that measuring light from the LED is irradiated to arbitrary points. The light source mounting bridge, lens holders, and stage mounting base were 3D-printed as well. Copper rod with a diameter of 9.5 mm was used for the support framing. The program for the measurement control/recording was written with Arduino IDE and in python with Visual Studio Code.

**Figure 1:**
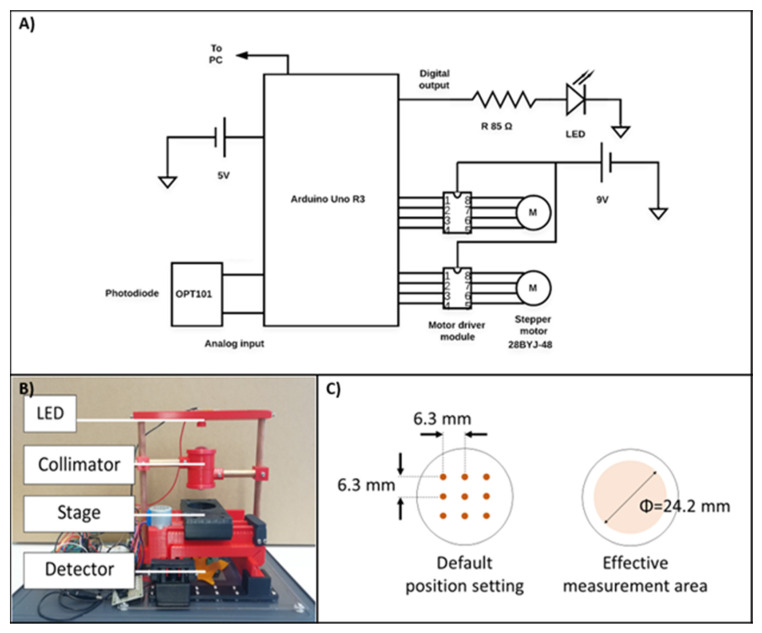
(**A**) Schematic electronic diagram of the measuring unit (OPT101: monolithic photodiode; Arduino Uno R3: microcontroller). (**B**) Picture of the semi-automatic system, with the major parts of the device: LED, Collimator, Stage, and photodiode detector, as mentioned in the materials and methods). (**C**) Position of the 9 spots over the 35 mm cell culture dish diameter, where the transmittance is measured.

As to the transmittance measurement, a single measurement is an average of 100 values read from the detector (placed under the sample stage) with the rate of 10 reads per second, per point (Figure 1: 9 points were measured at once). The analog output voltage of the photodiode in volts (V) as a signal of light intensity was converted to digital values by Arduino, to be processed by a computer. During the engineering cell sheet timeline, the transmittance was measured every other day, from the day the cells reached confluence (day 4 after initial seeding) and after replacing the culture media. The lid of the cell culture dish was removed, and the cell culture dish was placed on the device’s stage (Figure 1). The reference value was obtained by measuring the intensity of light coming through a cell culture dish that has the same amount of culture media, but with no cell sheet (blank sample). Collected values were converted to percent transmittance with respect to the value of the reference blank sample.

### 2.10. Measurement of the ASCCS Transmittance and Their Harvesting

Based on visual inspection and manipulator’s experience, the cell sheets, at different initial seeding density, were harvested at 8, 10, and 12 days, as mentioned above. The transmittance of the cell sheets was measured after replacing the culture media, to establish the standard curve for the different seedings. The 35 mm cell culture dishes were removed from the CO_2_ incubator and placed at room temperature, under a stereoscopic microscope. Twenty cell sheets were prepared per seeding (2.24 × 10^4^, 5.6 × 10^4^, 10.2 × 10^4^ ASC per cm^2^) and 4 cell sheets were harvested at the 8th, 10th, and 12th day after the initial seeding, to know if they can be harvested or not.

To harvest the multilayer cell sheets, we used a CellShifter membrane, with a 30 mm diameter (CellSeed, Inc., Tokyo, Japan) and forceps. The forceps were used to cut the edge of the cell sheet from the cell culture dish. The CellShifter was placed on the cell sheet. The culture media was removed, and the side of the cell sheet was wrapped over the edge of the CellShifter. By using the forceps, the cell sheet was lifted and placed in a new cell culture dish. Once placed in the cell culture dish, few drops of PBS were poured over the CellShifter. With the forceps, the CellShifter was lifted and detached from the cell sheet, which stayed attached to the cell culture surface. Half of the cell sheets were fixed in 10% neutral buffered formalin for immunohistochemistry staining or used for RNA extraction.

### 2.11. Statistical Analysis

All results are expressed as the mean ± standard deviation. The data were analyzed using a one-way ANOVA or a Student’s *t*-test. A *p* < 0.05 was considered statistically significant.

## 3. Results

**Figure 2:**
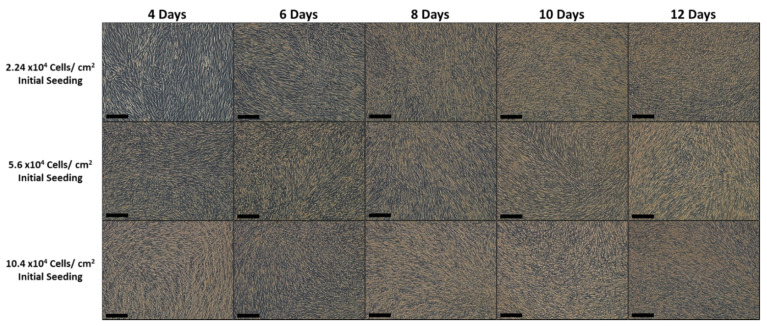
Picture showing the changes in the cell sheet morphology over time, up to 12 days, with 3 different initial ASC seeding. Notice the change of the ASC from a fibroblastic shape to a round shape (Scale bars are 200 µm).

**Figure 3:**
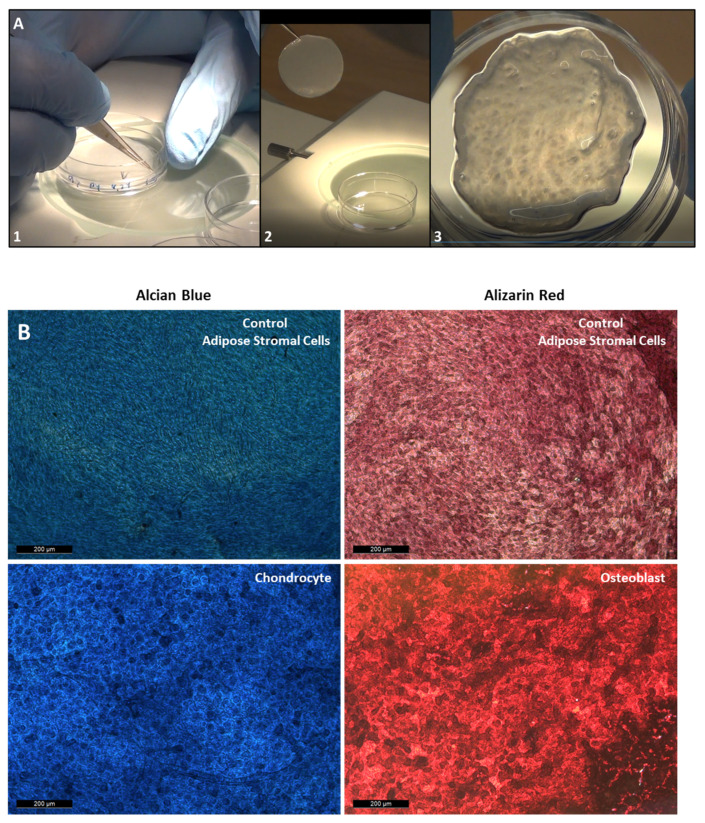
(**A**) Harvesting and lifting of the ASC cell sheets with a CellShifter. **1**: Cutting of the cell sheet edge, with forceps; **2**: lifting of the cell sheet using a cell shifter; **3**: transferred cell sheets in a new cell culture dish. (**B**) Dyeing of the ASC cells with Alcian blue and Alizarin Red after 2 and 3 weeks of differentiation into chondrocyte and osteoblast, respectively. (Scale bar are 200 µm).

Figure 2 shows how the cell sheets are engineered, using three different initial seeding of ASC. At day 4 after initial seeding, the cells from 2.24 × 10^4^, 5.6 × 10^4^ ASC/cm^2^ cell initial seeding were slightly over confluent, but the cells from the 10.2 × 10^4^ ASC/cm^2^ cell initial seeing were already forming a cell sheet. Every other day, pictures of the cell sheet formation were recorded. The density of the cells increased over time, forming strong and thick multilayer cell sheets. Cell sheets were harvested at the 8th, 10th, and 12th day. At day 8, even if it is difficult to translate in words the harvesting feeling, cell sheets from 2.24 × 10^4^ ASC/cm^2^ were weak and required more precision moves for their harvesting. For the 5.6 and 10.2 × 10^4^ ASC/cm^2^ were strong and easily harvested. From day 10 and beyond, for the three groups, the manipulators feel that the ASC were strong and easily mechanically harvested. In Figure 3A, the three steps for lifting the cell sheets are shown, as it is described in the material and methods section. The Figure 3B shows that the ASC used to engineer cell sheets can differentiate into chondrocyte and osteoblast cells.

**Figure 4:**
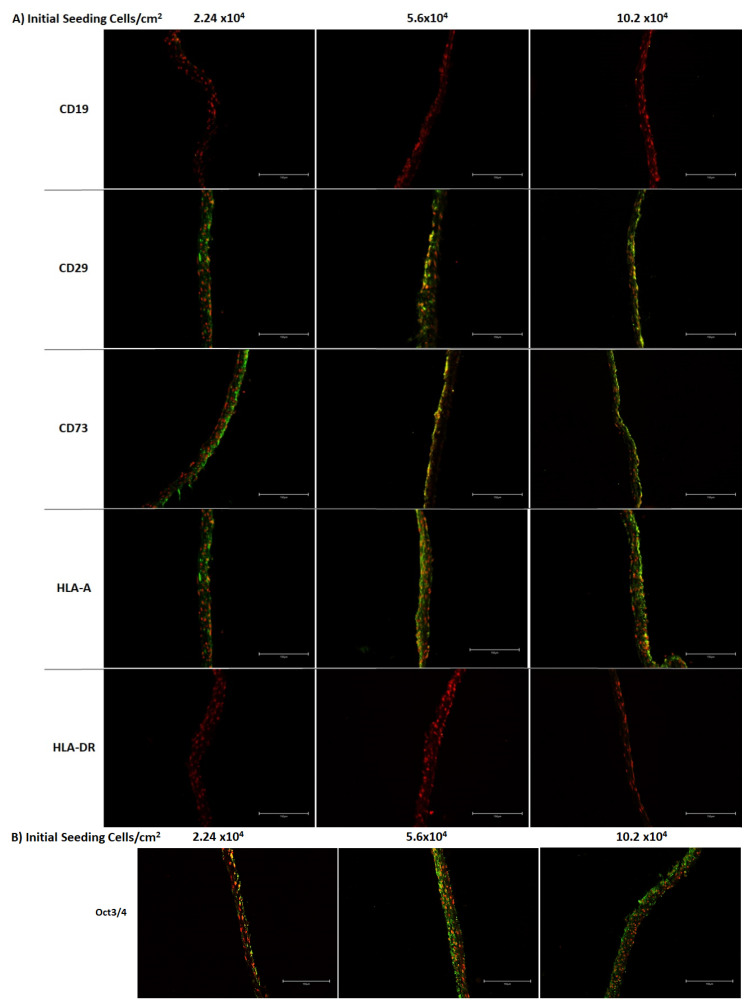
(**A**) Immunostaining on CD19, CD29, CD73, HLA-A, and HLA-DR was performed on the undifferentiated cell sheets harvested at 12 days, initially seeded with 2.24 × 10^4^, 5.6 × 10^4^, 10.2 × 10^4^ ASC per cm^2^ (scale bar 150 µm). (**B**) Immunostaining on 3/4 October was performed on the undifferentiated cell sheets harvested at 12 days, initially seeded with 2.24 × 10^4^, 5.6 × 10^4^, 10.2 × 10^4^ ASC per cm^2^ (scale bar are 150 µm).

In the Figure 4, the expression of ASC markers was verified in the preserved ASCCS, based on the criteria published by Dominici et al. [31]. The expression of CD29, CD73, and HLA-A were maintained until the 12th day, after the initial seeding. CD19 and HLA-DR were not detected in the ASCCS. In addition, we found that Oct3/4 protein, a stemness marker, is expressed well over the cell sheets.

**Figure 5:**
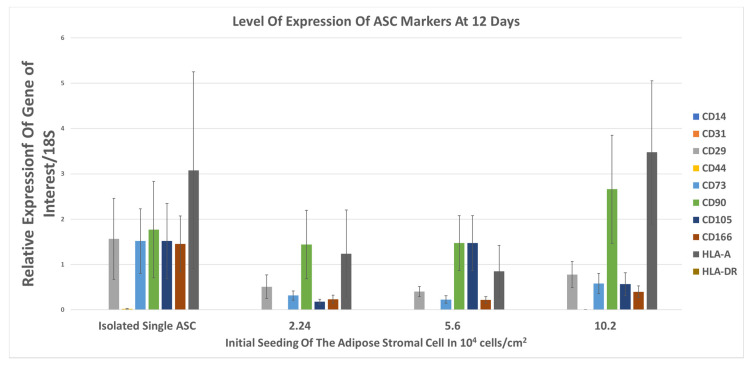
Relative level of expression of ASC markers, in the cell sheets compared to the isolated ASC, at 12 days (n = 3, mean ± SD).

The expression of mRNA of CD14, CD29, CD31, CD44, CD73, CD90, CD105, CD166, HLA-A, and HLA-DR were analyzed at 12 days. In Figure 5, isolated ASC express the CD29, CD73, CD90, CD105, CD166, and HLA-A mRNA, and CD14, CD31, and HLA-DR are not expressed. However, over time, the expression of CD29, CD73, CD105, CD166 decreased, on the three different groups of ASCCS. The expression of CD90 and HLA-A were maintained over time.

**Figure 6:**
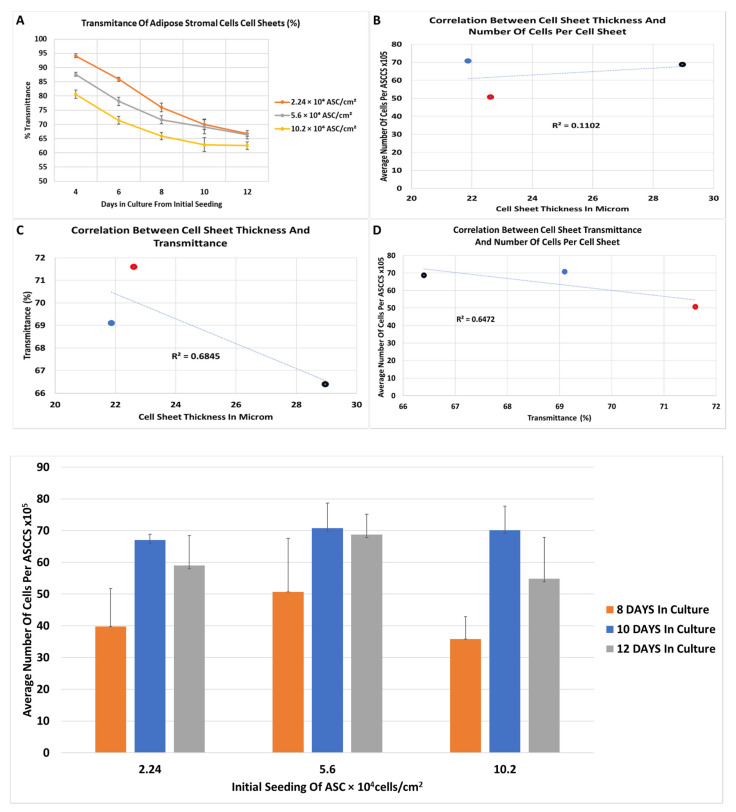
(**A**) Transmittance of the cell sheets at different initial cell density, over 12 days. 75% (Day 4: n = 20; Day 6: n = 20; Day 8: n = 20 except for the 10.2 × 10^4^/cm^2^ group where n = 16; Day 10: n = 16 for 2.24 × 10^4^/cm^2^ and n = 12 for 5.6 × 10^4^/cm^2^ and 10.2 × 10^4^/cm^2^; Day 12: n = 12 for 2.24 × 10^4^/cm^2^ and n = 8 for 5.6 × 10^4^/cm^2^ and 10.2 × 10^4^/cm^2^). *p*-value of <0.01 when compare same initial seeding between the days or when compared the 3 groups in the same day. No difference was measured at 12 days, between 2 and 5 × 10^5^ cells initial seeding. (**B**) It shows the non-correlation between the cell sheet thickness and the number of cells per cell sheets. (**C**) It shows the non-correlation between the cell sheet thickness and the loss of transmittance. (**D**) It shows the non-correlation between the loss of transmittance and the number of cells per cell sheet. (For (**B**–**D**): Cell sheets engineered with initial seeding with 5.6 × 10^4^/cm^2^, for 8, 10, and 12 days were studied to establish a correlation between the transmittance, thickness and the number of cells per cell sheets. Cell sheets used to determine the number of cells per cell sheet n = 6; cell sheets used to determine the cell sheet thickness n = 4; cell sheets to determine the transmittance are 20 for day 8, 12 for day 10, and 8 for day 12; dots in red represent the cell sheets harvest at day 8, dots in blue the cell sheets harvested on the 10th day, black dots are the cell sheets harvested on the 12th day). (**E**) It shows the content of DNA in whole ASCCS, using as a reference the dsDNA purified from isolated adipose stromal cells. For all groups, there is a significant difference between 8 days vs 10 days and 8 days vs 12 days (*p* < 0.05), and no significant difference between the 10 and 12 days in culture (n = 6, mean ± SD).

Figure 6 reports the decrease in transmittance overtime of the cell sheets. As expected, the transmittance at the 4th day was higher for the lowest initial cell seeding at 2.24 × 10^4^ (94.1% ± 0.7) compared to the 5.6 × 10^4^ (87.6% ± 0.74) and 10.2 × 10^4^ ASC (80.58% ± 1.48) per cm^2^. The trend of the transmittance decrease, over time, was similar in between the three groups, and the slope of the transmittance curve decreased to reach a plateau in the transmittance (Figure 6A). Based on the harvesting of the cell sheets related with the manipulators experience, undifferentiated stratified cell sheet can be harvested and lifted with ease when the transmittance reached 75% or lower.

As the transmittance of the cell sheets decreased over time, we expected that the thickness of a cell sheet and number of cells inside will increase proportionally. To confirm this hypothesis, we measured the average thickness and the average number of cells per cell sheets, at 8, 10, and 12 days, for cell sheets engineered with 5.6 × 10^4^/cm^2^ initial seeding. It is very difficult to determine the thickness of a cell sheet because the thickness is not homogenous over the sample. We decided to estimate the average thickness of the ASCCS by measuring the thickest and the thinnest cross section of H&E of the cell sheets, randomly. To determine the number of cells per cell sheet, a different approach was used. In general, the cell sheet could be digested with an enzyme, and the number of cells per cell sheet could be counted with the trypan blue methodology. However, ASCCS are very difficult to digest with dispase, trypsin and another enzyme, and it was observed in other laboratories [30]. We decided to isolate the genomic DNA from the entire cell sheets and to measure the content of the double strand DNA (dsDNA). The dsDNA from isolated ASC were used as a reference to estimate the number of cells per ASCCS. In Figure 6B–D, no correlation was found between the cell sheet thickness, the number of cells per cell sheet and the transmittance. Figure 6E shows that the number of cells per cell sheets reached their maximum at 10 days after initial seeding. In Figure 6E, for the three different seeding groups, there is a significant increase of DNA content between the 8 days in culture with 10 days in culture, and 8 days in culture with 12 days in culture. The ASCCS reached the maximum number of cells at 10 days because the number of cells does not increase over time significantly at 12 days.

**Figure 7:**
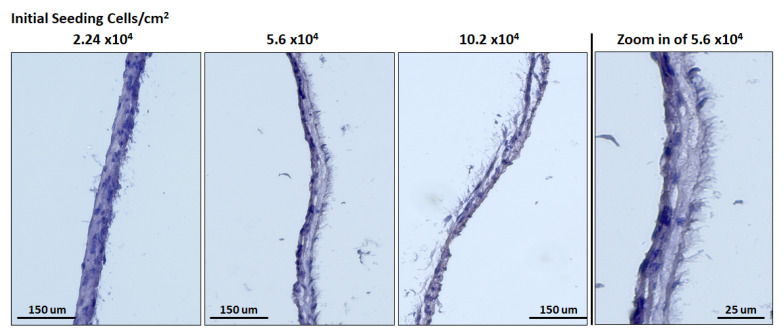
H&E on the undifferentiated cell sheets harvested at 12 days, initially seeded with 2.24 × 10^4^, 5.6 × 10^4^, 10.2 × 10^4^ ASC per cm^2^. The figure on the right shows a zoom in of the cell sheet engineered with 5.6 × 10^4^ per cm^2^, and microvilli can be observed on the apical side of the cell sheets.

After performing Hematoxylin & Eosin dying on the cell sheet, the formation of “microvilli” was noticed, for all the ASCCS (Figure 7). The function of these microvilli is unknown, and at the best of our knowledge, was not reported. They could be the brighter areas we noticed over the cell sheet, during the cell culture (Figure 2) and could be responsible for the decrease of the transmittance as their surface seemed to increase over time.

## 4. Discussion

Using different culture media, we succeeded to engineer multilayer undifferentiated adipose cell sheets, and maintained the expression of ASC markers (CD29, CD73, HLA-A) or not (CD19, HLA-DR). The expression of Oct4 was detected over the cell sheets, confirming the preservation of the cell’s stemness [32,33,34]. The time for harvesting will vary based on the type of seeded cells (bone marrow cells, dental pulp stem cells, oral mucosal epithelial cells, induced pluripotent stem cells [35,36,37,38], myocardiac cell sheet, osteoblast cell sheet, epithelial cell sheet [11,39,40]), the nature of the culture media, the rate of differentiation, and the formation of the stratified cells sheets. We decided to use the different levels of transparency of engineered cell sheet with adipose derived cells to develop a device that will measure the transmittance of light through the cell sheets.

In this study and for the first time, we have developed and used an affordable device to measure the transparency of the adipose stem cells cell sheets. The support design is based on a 35 mm cell culture dish, but other type of cell culture dish could be tested, with a specific engineered support such as for 60 mm cell culture dish or 6 well plate as long as the stage is adapted to the cell culture dish. The principle is to project white light from the top of the cell culture dish and measure the percentage of light going through the cell sheet. Like any other materials, the absorption coefficient of a cell sheet should be dependent on wavelength of transmitting light, thus the transmittance is by nature expected to be influenced by the wavelength of the light source. However, as a device that provides relative values of transmittance to indicate the degree of cell sheet maturity, wavelength used for the measurement can be arbitrary selected. If different wavelength is used for measurement, a specific standard curve is to be created accordingly. We have demonstrated that we can measure the transmittance of the cell sheets and utilize the obtained values as an indicator of the timing for harvesting. A cell sheet can be harvested when cultured cells create, during their maturation process, a tight and strong intercellular connection which contributes to mechanical integrity of a sheet structure [8,41]. Since the degree of maturation of cell sheets’ intercellular connection is quantifiable only by invasive manners, the timing of harvesting is to be determined by operator’s experience or morphological observation, which is subjective and requires experience to correlate morphological appearance with cell sheet maturity. In the field of three-dimensional (3D) cell culturing or tissue engineering, it is generally known that the light-penetrating properties of the sample diminishes as cells grow and form a stratified structure. In fact, low transparency is one of the challenges when studying a 3D cell culture or tissue structure because ordinary imaging analysis methods that require certain amount of reference light to be penetrated could hardly be used [42,43]. To put it the other way around, transmittance of light could potentially be a quantitative measure that reflects maturity of a cell sheet, thus indicating the timing of harvesting. The decrease in transmittance of a 3D cell structure is primarily due to scattering of light at the surface and inside the sample, which can be attributed to thickness of samples, high cell density and development of extra-cellular matrix [44]. Our first hypothesis was that the decrease of the transmittance measured over time was proportionally correlated with the increase of the cell sheet thickness. Unfortunately, no correlation was found between the ASCCS thickness, and the transmittance decrease overtime. We then suggested that the increase in cell density in the ASCCS could explain the transmittance decrease. However, we did not find a correlation between the 33.2%, 33.4%, and 37.47% of transmittance decrease for the 2.24 × 10^4^, 5.6 × 10^4^, 10.2 × 10^4^ ASC per cm^2^, respectively, at the 12 days and the estimate number of cells present in the ASCCS.

Another potential explanation for the decrease of the transmittance is the development of extracellular matrix (ECM) and abundance of cell-cell/cell-matrix interconnecting proteins. A multilayered cell sheet possesses rich ECM [45,46], and transparency diminishes as fiber density increases [47]. The structure and orientation of fibrous component in ECM also affect the light scattering pattern. For instance, cornea is known as one of the tissues that has multilayer structure consists of cells and ECM, yet optically transparent. One of unique features of corneal tissue that contributes to its transparency is highly regulated collagen fibril, one of the primary components of ECM, and its organized spacing in stromal ECM [48,49]. On the other hand, study on orientation of collagen reveals that its spatial randomness causes the inhomogeneous pattern of refractive index in the tissue, which increases the degree of scattering [50,51]. It has been shown that transformation of a MSC cell sheet from 2D monolayer to 3D multilayer upon detachment from cell culture dish resulted in morphological rearrangement of actin cytoskeleton from unidirectional to multi-directional alignment, which makes a 3D multilayer cell sheet opaquer [52]. Furthermore, MSC cell sheets fabricated with supplementation of fetal bovine serum (FBS) and human platelet lysate (hPL) exhibited unidirectionally and multi-directionally aligned actin cytoskeletal structures, respectively, where cell sheets with multi-directional actin structure had relatively opaque appearance [53]. It is reasonable to associate the opaqueness of these cell sheets to its multi-directional, random orientation of actin cytoskeleton, as it augments the degree of scattering of irradiated light. Moreover, 2D-to-3D transformation of a cell sheet showed enhanced cell–cell and cell–matrix interaction, resulting in upregulation of B-catenin, connexin 43, and Integrin B1 [52], all of which creates strong cell–cell connection. Therefore, the decrease in transmittance could be attributed to the maturation of complex and dense ECM and cell-cell connecting network, as well as multi-directional alignment of cytoskeletal actin as the cell sheets develop 3D structure. However, the phenotypic characteristics of mesenchymal stem cells cultured in vitro, including various growth factors, are dependent on initial seeding cell density [54,55], which provides potential explanation why transmittance values for samples with initial seeding density of 10.2 × 10^4^/cm^2^ reached different value at the plateau from other two conditions.

Needless to say, many other factors should be taken into account when considering a relationship between 3D structure formation and optical transmitting property, and it should also vary depending on the type of tissue. For example, oral mucosal epithelial cells sheets are formed of four to five layers and they are transparent [56,57], while the transparency of the muscle-derived cell sheet, forming six to seven layers, seems very low [58,59]. Of course, we do not have the value of the transmittance of these different types of cell sheet, and there is no peer-review reporting the measurement of cell sheet transmittance to be used as a reference. However, we observed the apparition of beige spots early in the ASC culture timeline, which increased in size, forming larges area when ASC are forming ASCCS. Those beige areas formed over the cell sheets, on the upper layers of the cell sheets. They seem to be opaque which could be another explanation why the transmittance of the cell sheet decreased over time, without thickness and number of cells change. Hematoxylin and Eosin dye showed the presence of microvilli structure over the apical side of the undifferentiated cell sheets. Such structure was already reported when ASC were cultured and undifferentiated [60,61]. The appearance of the microvilli suggests the polarization of the cell sheets, which occur during the formation of tissue or to maintain the tissue architecture. The polarization could be due to different factors such as the nature of the extracellular matrix, the architecture of the cytoskeleton (which is related also with the composition of the extracellular matrix), mechanical forces [62,63,64,65,66]. It is possible that the undifferentiated cell sheet tends to differentiate or have a high potential to differentiate into epithelium [45]. We understand that additional experiments will be necessary and specific culture media will used to differentiate the cell sheets into a polarized epithelium [9].

We also ensured about the cell sheet stemness, for the three different initial seeding at 12 days. The maintenance of the stemness properties is critical if the ASCCS are to be transplanted on organs, and favorized their potential differentiation or their support in the treatment of the diseases [56,59,67]. The expression of ASC markers decreased compared to the initial isolated ASC, but they are still expressing, indicating that ASCCS could still differentiate into different type of cells, before or after transplantation [8]. In addition, the absence of expression of the HLA-DR is important because of lowering the risk of immune-rejection, for allogenic transplantation and allowing the mass production of ASCCS [68].

We understand that our study focused on a specific cell sheet, engineered with ASC, but for the first time, we are reporting a device measuring the transmittance of multilayered cell sheets, and more particularly the ASCCS. This device could address the needs in the increasing cell sheet technology field and could help inexperienced people in the field of cell sheet to determine when a cell sheet is ready for harvesting, in well-defined cell culture conditions (culture media, type of cells, initial seeding, etc.). Moreover, additional studies will be necessary to study the transmittance of different type of cell sheets, that can be engineered with different sources of cells: cardiac, corneal, dental, epithelial [30,35,69,70,71].

## 5. Conclusions

Stratified cell sheets, using adipose stromal cells, were engineered and they maintained their differentiation properties. We have developed an affordable device, to measure the transmittance of multilayer cell sheets using a light emitting diode and a photodiode, in a non-invasive approach. Cell sheet transmittance was measured overtime and correlated with the cell sheets maturation. Based on a standard transmittance curve, recorded transmittance could be used to estimate the cell sheets harvesting time. In addition, this non-invasive cell sheet transmittance measurement could be included in cell sheet quality control, for translational applications.

## 6. Patent

A provisional patent application Serial No.63/205,757 for “Device and Related Methods for Measuring Properties of Cell Sheets and Tissues” is the result from the work reported in this manuscript.

## Figures and Tables

**Table 1:** List of the primers used for the real-time PCR.

Gene of Interest	Gene ID	Forward Primer	Reverse Primer
18S	NR_003286.4	CGGCTACCACATCCAAGGAA	GCTGGAATTACCGCGGCT
HLA-A	NM_002116	AGATACACCTGCCATGTGCAGC	GATCACAGCTCCAAGGAGAACC
HLA-DR	NM_002124	GAGCAAGATGCTGAGTGGAGTC	CTGTTGGCTGAAGTCCAGAGTG
CD14	NM_000610.4	CAACCTAGAGCCGTTTCTAAAGC	GCGCCTACCAGTAGCTGAG
CD29	NM_033667	GGATTCTCCAGAAGGTGGTTTCG	TGCCACCAAGTTTCCCATCTCC
CD31	NM_000442	AAGTGGAGTCCAGCCGCATATC	ATGGAGCAGGACAGGTTCAGTC
CD44	NM_000610.4	CCAGAAGGAACAGTGGTTTGGC	ACTGTCCTCTGGGCTTGGTGTT
CD73	NM_002526.4	CTCCTCTCAATCATGCCGCT	CCCAGGTAATTGTGCCATTGT
CD90	NM_001311160.2	GAAGGTCCTCTACTTATCCGCC	TGATGCCCTCACACTTGACCAG
CD105	NM_001278138.2	CGGTGGTCAATATCCTGTCGAG	AGGAAGTGTGGGCTGAGGTAGA
CD166	NM_001243280.2	TGGCAATATCACATGGTACAGGAA	CCAGGGTGGAAGTCATGGTATAGAG

## Data Availability

Not applicable.
